# Supplementary material for: Association of single nucleotide polymorphisms with dyslipidemia in antiretroviral exposed HIV patients in a Ghanaian population: A case-control study
Source: PLoS One. 2020 Jan 13;15(1):e0227779. doi: 10.1371/journal.pone.0227779 (PMC6957303; doi:10.1371/journal.pone.0227779)
Supplement: S1 Table — (DOCX) [file pone.0227779.s002.docx]

**Supplementary data**

**Table 1: Demographic and clinical characteristics of study participants based on antiretroviral therapy**

| **Demography** | **Total**  **n=289** | **NNRTI Based ART**  **n=279** | **PI-Based ART**  **n=10** | **p value** |
| --- | --- | --- | --- | --- |
| Age (years) | 39.74±10.12 | 39.86±9.955 | 36.20±11.82 | 0.2569 |
| Sex |  |  |  | 0.6929 |
| Male | 56 (19.4%) | 55(19.8%) | 1(1.0%) |  |
| Female | 232 (80.6%) | 223(80.2%) | 9(99.0%) |  |
| Duration of Treatment (years) | 4.32±2.877 | 4.36±2.85 | 3.20±2.43 | 0.2092 |
| Blood Pressure |  |  |  |  |
| Systolic (mmHg) | 114.10±16.50 | 114.00±16.44 | 115.60±19.00 | 0.7638 |
| Diastolic (mmHg) | 81.08±7.92 | 80.93±7.88 | 85.30±8.71 | 0.0865 |
| Weight (Kg) | 62.89±13.24 | 57.75±18.75 | 63.07±13.02 | 0.2122 |
| Height (m) | 1.59±0.14 | 1.59±0.14 | 1.61±0.06 | 0.7221 |
| BMI (kg/m^2^) | 32.19±17.82 | 32.28±18.16 | 29.92±5.71 | 0.6825 |
| Lipid parameters |  |  |  |  |
| Total Cholesterol (mmol/L) | 4.41±0.99 | 4.34±0.96 | 6.09±0.56 | **0.0001** |
| Triglycerides (mmol/L) | 1.48±0.72 | 1.43±0.69 | 2.61±0.65 | **0.0001** |
| HDL-Cholesterol (mmol/L) | 0.99±0.53 | 0.99±0.54 | 0.96±53 | 0.8895 |
| LDL-Cholesterol (mmol/L) | 3.01±1.18 | 2.95±1.16 | 4.60±0.55 | 0.0001 |
| Coronary Risk | 7.92±3.72 | 7.84±3.78 | 9.86±0.27 | 0.274 |
| Dyslipidaemia n (%) | 89(30.9%) | 88(31.7%) | 1(10.0%) | **0.0002** |
| Hypertriglyceridemia | 26(9.0%) | 23(8.3%) | 3(30.0%) | **0.0001** |
| Hypercholesterolemia | 104(36.1%) | 103(37.1%) | 1(1.0%) | **<0.0001** |
| Low HDL-C | 197(68.4%) | 189(67.9%) | 8(80.0%) | **0.076** |

**^a^Fisher exact text, HDL-C=High Density Lipoprotein, LDL=Low Density Lipoprotein, PI=Protease Inhibitor, ART=Antiretroviral Therapy, p<0.05=Statistically Significant**.
