# Supplementary material for: Association of single nucleotide polymorphisms with dyslipidemia in antiretroviral exposed HIV patients in a Ghanaian population: A case-control study
Source: PLoS One. 2020 Jan 13;15(1):e0227779. doi: 10.1371/journal.pone.0227779 (PMC6957303; doi:10.1371/journal.pone.0227779)
Supplement: Attachment — Submitted filename: Response to Reviewers comments.docx [file pone.0227779.s004.docx]

**Response to Editorial comments**

**Dear Editor-In-Chief**

**Manuscript tittle: Association of Single Nucleotide Polymorphisms with Dyslipidemia in Antiretroviral Exposed HIV Patients in a Ghanaian population**

**Article # PONE-D-19-22071**

**We sincerely thank the reviewers for the truly helpful comments to improve the quality of our manuscript. We have read through carefully, please find our detailed responses below each specific point**

Review Comments to the Author

Reviewer #1:

**Comment:** The author has addressed all my questions. I don't have other major concerns. However, there is still a few minor issues. For example, in the introduction, "Low-density lipoprotein receptor (LDLR), positioned on chromosome 19p13.2 plays a significant role lipoprotein metabolism by mediating the uptake of cholesterol through the binding and subsequent cellular uptake of apolipoprotein-E and B- constituting lipoproteins." should be "Low-density lipoprotein receptor (LDLR), positioned on chromosome 19p13.2 plays a significant role in lipoprotein metabolism by mediating the uptake of cholesterol through the binding and subsequent cellular uptake of apolipoprotein-E and B- constituting lipoproteins." so the author needs to proofread the whole manuscript carefully.

**Response: Thank you for the comment. Section has been revised and manuscript has reviewed by an English native speaker**

Reviewer #2:

The revised version of the manuscript by Acheampong et al is still very far from being suitable for publication, for the next reasons:

**1. Comment**: The authors did not satisfactorily answer the overall major concern regarding the design of the study and the rationale to include HIV-seronegative individuals. This is a study about the influence of several SNPs in the presence of dyslipidemia in HIV-patients on HAART. So, unless there is a very clear and strong reason as I explained in my first revision, HIV-seronegative individuals must be excluded from the study.

I strongly recommend the authors to exclude the 104 HIV-seronegative individuals from the study and reanalyse the data again only with the 289 HIV-patients.

**Response: Thank you for the comment, the authors are grateful. We have excluded the 104 HIV sero-negative individuals from the study population and then reanalyse the dataset for the 289 HIV patients**

2.**Comment**: The design of the study is not clear at all and authors must clarify this. Is this a case-control study? (in my opinion it is). I already explained in my first revision who are the cases and who are the controls in a case/control study. If the authors do not consider that this is a case/control study, then the use of the terms “study groups”; “HIV+Dys+”; “HIV+Dys-“ is OK but all mention to “cases” and “controls” must be deleted from the manuscript. I strongly recommend the help of a statistician.

**Response: Thank you for the comment. We believed that base on the reviewer comment and it is case-control study, section has been revised. We have considered the study as case-control and therefore the terms “HIV+Dys+” and “HIV+Dys-” has been removed from the manuscript. A statistician was involved in analysing the data.**

3. **Comment:** The way data are presented in tables 4 and 5 (logistic regression with diyslipidemia and with individual lipid abnormalities) and in figures 2 and 3, is incomprehensible. As the authors explain, the odds ratios shown in these tables and figures are referred to the “control” HIV-seronegative population. This is very difficult to understand. The clearest way to show evidence for association between the genotype of the different SNPs and the existence of dyslipidemia, is to calculate the odds ratios of having dyslipidemia in carriers of the minor homozygous genotype for each of the SNPs, taking the other individuals (carriers of the major homozygous and of the heterozygote genotypes) as reference (OR=1).

**Response: Thank you for the comments. Tables 4 and 5 have been revised based on the reviewer’s comment. Figures 2 and 3 have removed. We have reanalysed the data by calculating the odd ratios taking into consideration the combination of the carriers of the major homozygous and the heterozygote genotypes as referent**

4**.Coment**: The manuscript is still plagued with grammatical errors, sentences difficult to understand and words not appropriate, etc…I strongly recommend that the whole manuscript is revised by a native English speaker

**Response: Thank you for the comment. Our manuscript has been reviewed and revised by two native English speakers.**
